# Supplementary material for: Community knowledge, attitude and practices to SARS-CoV-2 disease 2019 (COVID-19): A cross-sectional study in Woldia town, Northeast Ethiopia
Source: PLoS One. 2021 Apr 20;16(4):e0250465. doi: 10.1371/journal.pone.0250465 (PMC8057572; doi:10.1371/journal.pone.0250465)
Supplement: Attachment — Submitted filename: Response to reviewers.docx [file pone.0250465.s001.docx]

**Response to Editor’s and reviewers comments**

**Authors’ response to Editor’s comments**

1. Please ensure that you refer to Figure 2 in your text as, if accepted, production will need this reference to link the reader to the figure.

**Authors’ response to comment 1**

- In the previous submitted manuscript Fig. 2: is corrected with the correct guideline form of PLOS ONE style as Fig 2. in the corrected manuscript.

2. Your ethics statement should only appear in the Methods section of your manuscript. If your ethics statement is written in any section besides the Methods, please move it to the Methods section and delete it from any other section. Please ensure that your ethics statement is included in your manuscript, as the ethics statement entered into the online submission form will not be published alongside your manuscript.

**Authors’ response to comment 2**

- The ethics statement is appeared only in the method section and removed from other part of the corrected manuscript

3. Please provide additional details regarding participant consent. In the ethics statement in the Methods and online submission information, please ensure that you have specified 1) whether consent was informed, and 2) what type you obtained (for instance, written or verbal, and if verbal, how it was documented and witnessed).

**Authors’ response to comment 3**

1. We have obtained a written consent from the respondents. The respondents’ willingness to give the data is confirmed by their signature as indicated below on page 3 and 4. The written consent was prepared both by using English and Amharic version.

4. Please amend the title either on the online submission form or in your manuscript so that they are identical.

**Authors’ response to comment 4**

- The corrected title of our research title is **“Community knowledge, attitude and practices to** SARS-CoV-2 **disease 2019 (COVID-19):** A cross-sectional study **in Woldia town, Northeast Ethiopia”.** This corrected title is also written in the previous manuscript based on your comment**.**

5. Thank you for providing the following response regarding Figure 1 of your manuscript: "Figure 1 indicated in the manuscript is not copyrighted. We prepare the map by using GIS software and so it does not need any permission. If our reason is not acceptable we can remove from the manuscript."
   Please clarify the source of the data used to generate your map in GIS.

**Authors’ response to comment 5**

- The source of the data used to generate our map in GIS is projected coordinate system taken from North Wollo shape files. Source link: [www.diva-gis.org/gdat](http://www.diva-gis.org/gdat)

6. Please ensure that your manuscript meets PLOS ONE's style requirements, including those for file naming.

**Authors’ response to comment 6**

- We have followed the author guideline of the PLOS ONE and we have corrected the given comments in the revised manuscript accordingly.

7. Please note that whilst COVID-19 refers to the name of the disease, the name of the virus that causes it is SARS-CoV-2.

**Authors’ response to comment 7**

- In the corrected manuscript we have incorporate COVID-19 as the disease and SARS-CoV-2 as the virus that cause the disease COVID-19. Novel corona virus is completely substituted with SARS-CoV-2

8. We suggest you thoroughly copyedit your manuscript for language usage, spelling, and grammar.

**Authors’ response to comment 8**

- The authors tried to correct grammar, spelling, punctuation and other important mistakes in the manuscript.

9. Please ensure you have discussed any potential limitations of your study in the Discussion, including study design, sample size and/or potential confounders.

**Authors’ response to comment 9**

- We have tried to discuss and avoid limitation of discussion. Limitation of the study part is now included in the corrected manuscript. We have also clearly indicated the design and the sample size is determined that represents the study population.

10. In your Methods section, please provide additional information about the participant recruitment method and the demographic details of your participants. Please ensure you have provided sufficient details to replicate the analyses such as a description of how participants were recruited.

**Authors’ response to comment 10**

- The study participants are recruited by using random sampling technique whose age is 18 and above.

11. If you developed a questionnaire as part of this study and it is not under a copyright more restrictive than CC-BY, please include a copy, in both the original language and English, as Supporting Information.

**Authors’ response to comment 11**

- The developed questionnaire is written both in the native language (Amharic) and English version. It is attached at the end on page 11 below.

12. Please amend your current ethics statement to include the full name of the ethics committee/institutional review board(s) that approved your specific study.

**Authors’ response to comment 12**

- Woldia University ethics research committee has given us Ethical approval clearance to collect the required data from the respondents.

13. Please clarify the sources of funding (financial or material support) for your study. List the grants or organizations that supported your study, including funding received from your institution.

**Authors’ response to comment 13**

- The authors received no specific funding for this work.

14. Please complete your Competing Interests on the online submission form to state any Competing Interests.

**Authors’ response to comment 14**

- The authors have declared that no competing interests exist.

**Authors’ response to Reviewer’s comments**

1. Is the manuscript technically sounds, and do the data support the conclusions?

**Authors’ response to comment 1**

- We have included the information that has been given by the reviewer by including some data into the conclusion part and we hope it make the manuscript technically sound.

2. Has the statistical analysis been performed appropriately and rigorously?

**Authors’ response to comment 2**

- The data were analyzed and performed appropriately by using chi-square test in SPSS software to see the association of socio-demographic characteristics of the respondents to their knowledge, attitude and practices towards COVID-19.

3. Have the authors made all data underlying the findings in their manuscript fully available?

**Authors’ response to comment 3**

- All the data underlying the findings in their manuscript fully available in the manuscript.

4. Is the manuscript presented in an intelligible fashion and written in standard English?

**Authors’ response to comment 4**

- We have seen the grammar, spelling and punctuation mistakes and we have corrected. The language in the submitted articles is clear, correct, and unambiguous and we improved the readability.

5. I am not sure that the mention made by authors that there is no scientifically vaccines holds true since a lot of vaccine candidates are in late phase trials already.

**Authors’ response to comment 5**

- Until this research data analyzed, there is no scientifically vaccines holds true since a lot of vaccine candidates are in late phase trials already. But, now there are some vaccines which are scientifically accepted.

6. Explain what does the term “Kebeles"

**Authors’ response to comment 6**

- “Kebelle” means a small administrative unit in a town.

7. The abstract needs to be written completely as has incomplete sentences. The conclusion of the abstract does not seem appropriate.

**Authors’ response to comment 7**

- The incorrect sentences in the abstract are re-written again.

8. Authors have mentioned the Novel Corona Virus as "Nobel" Corona virus at multiple places.

**Authors’ response to comment 8**

- In the first submitted manuscript “nobel” corona virus is written instead of novel corona virus because of type error. Now it is corrected and it is replaced with SARS-CoV-2.

9. The data presented by the authors describes about the socio-demographics of the study population but does not elaborate how the KAP are correlated. The authors should elaborate on how the KAP are being affected by the education, occupation or age of the study population.

**Authors’ response to comment 9**

- We have done an association between socio-demographic characteristics and KAP by using chi-square test, but we have not done correlation test.

10. The authors have compared their study with other studies in other countries but comparisons need to be elaborated upon and need data from other studies as well. For example the authors mention - The findings of this study are not in agreement with the study done in Malaysia. No data is provided about the Malaysian study.

**Authors’ response to comment 10**

- The finding of this study is compared with a study done in Malaysia and the data is included (83.1%) in the corrected manuscript.

**ቃለመጠይቅ**

**ወልዲያ ዩኒቨርሲቴ**

**ተፈጥሮና ቀመር ሳይንስ ፋካልቴ**

**ባዮሎጅ ትምህርት ክፍል**

**መመሪያ**፡ የዚህ ቃለመጠይቅ ዋና አላማ በኮቪድ-19 በሽታ የወልድያ ከተማ ማህበረሰብ ስላለዉ እዉቀት፡ አመለካከት ና ትግበራ የዳሰሳ ጥናት ለማድረግ ነዉ፡፡ ይህ ቃለመጠይቅ የጥናቱ ተሳታፊዎችን ፆታ፤ እድሜ፤ ጋብቻ፤ ትምህርት ደረጃ ና የስራ ሁኔታ ስለ ኮቪድ-19 በሽታ ያላቸዉን አጠቃላይ ::የዚህ ጥናት መሳካት የጥናቱ ተሳታፊዎች የሚሰጡት ትክክለኛ መረጃ ለጥናቱ ዉጤት በጣም አስፈላጊ ነዉ፡፡ስለዚህ ለተጠየቁት ጥያቄዎች ትክክለኛ መልስ በመስጠት ጥናቱ የተሟላ እንዲሆን ያለዉ አስተዋፅዎ ከፍተኛ ነዉ፡፡ በመጨለሻም ለመጠይቁ ፈቃደኛ በመሆንዎ ከልብ እናመሰግናለን፡፡

**ክፍል አንድ፡-ለሚከተሉት ጥያቄዎች ለሚመርጡት መልስ የራይት ምልክት በተቀመጠዉ ሳጥን አስቀምጡ፡፡**

| **የጥናቱ ተሳታፊዎች ዲሞግራፊ** | | |
| --- | --- | --- |
| ተ.ቁ | ተለዋዋጭ |  |
| 1 | እድሜ | ________ |
| 2 | ፆታ | ወ ሴ |
| 3 | የጋብቻ ሁኔታ | ያላገባ/ች |
|  |  | ያገባ/ች |
|  |  | የፈታ/ች |
|  |  | ባሏ የሞተባት |
| 4 | የትምህርት ደረጃ | ማንበብና መፃፍ የማይችሉ |
|  |  | ማንበብና መፃፍ የሚችሉ |
|  |  | የመጀመሪያ ደረጃ ትምህርት ያጠናቀቀ |
|  |  | ሁለተኛ ደረጃ ትምህርት ያጠናቀቀ |
|  |  | መሰናዶ ወይም ቴክኒክ ትምህርት ያጠናቀቀ |
|  |  | ዲፕሎማ |
|  |  | የመጀመሪያ ዲግሪ |
|  |  | ሁለተኛ ዲግሪ |
| 5 | የስራ ሁኔታ | ቅጥረኛ |
|  |  | ያልተቀጠረ |
|  |  | ጡረተኛ |
|  |  | ነጋዴ |

| **የጥናቱ ተሳታፊዎች በኮቢድ-19 ላይ ያላቸዉ የእዉቀት፡ አመለካከት ና ትግበራ** ሁኔታ | | |
| --- | --- | --- |
| 6 | ስለኮቢድ-19 መረጃ የተገኘበት መንገድ | በሬድዮና ቴሌቪዢን |
|  |  | ማህበራዊ ሚዲያ |
|  |  | ከጓድኛ |
| 7 | የኮቢድ-19 መተላለፊያ መንግድ | በመሳልና በማስነጠስ |
|  |  | ኮቢድ-19 ካለበት ሰዉ ጋር ባለ ንክኪ |
|  |  | በኮቢድ-19 በተበከለ እቃ አማካኝነት |
| 8 | የኮቢድ-19 በሽታ ምልክቶች | ትኩሳት |
|  |  | ደረቅ ሳል |
|  |  | ማስነጠስ |
|  |  | ለመተንፈስ መቸገር |
| **9** | ባህላዊ ዘዴዎችን መጠቀም (ነጭ ሽንኩርት፤ ጅንጅብል፤ ፌጦ) ኮቪድ-19 ለመከላከል) ይጠቅማል | አዎ |
|  |  | አይደለም |
| **10** | በኮቪድ-19 የተያዘ ሰዉን ከጤነኛዉ መለየት ፍቱን የሆነ የኮቪድ-19 ስርጭት ለመቀነስ ያገለግላል | አዎ |
|  |  | አይደለም |
| **11** | ኮቪድ-19 መቆጣጠር ይቻላል | አዎ |
|  |  | አይደለም |
| **12** | መንግስት ኮቪድ-19 ለመቆጣጠር በቂ የሆነ የመከላከል እርምጃ አከናዉኗል | አዎ |
|  |  | አይደለም |
| **13** | ጡት ማጥባት ኮቪድ-19 ከናት ወደ ልጅ ያስተላልፋል | አዎ |
|  |  | አይደለም |
| **14** | ህፃናት ለኮቪድ-19 ተጋላጭ አይደሉም | አዎ |
|  |  | አይደለም |
| 15 | ሁልጊዜ እጅን በዉሃና በሳሙና እታጠባለዉ | አዎ |
|  |  | አይደለም |
| 16 | ሁልጊዜ እጅን በሳኒታይዘር አፀዳላዉ | አዎ |
|  |  | አይደለም |
| 17 | ስስል አፌንና አፍንጫየን እሸፍናለዉ | አዎ |
|  |  | አይደለም |
| 18 | አይኔን፤አፍንጫየን ና አፌን አልነካም | አዎ |
|  |  | አይደለም |
| 19 | ማህበራዊ ፈቀቅታን እተገብራለዉ | አዎ |
|  |  | አይደለም |
| 20 | ህዝብ በተሰበሰበበት ቦታ የአፍነጫና አፍ መሸፈኛ እጠቀማለዉ | አዎ |
|  |  | አይደለም |
| 21 | የስራ ቦታየን ሁልጊዜ አፀዳለዉ | አዎ |
|  |  | አይደለም |

**ክፍል ሁለት:- የመንግስት ኮቪድ-19 የመከላከል ትግበራ በማህበረሰቡ የሚታየዉ አፈፃፀም መልስ የሆነዉን በተሰጠዉ ሳጥን ዉስጥ የራይት ምልክት አስቀምጥ/ጭ**

መመዘኛ;- 5= በጣም እስማማለዉ; 4 =እስማማለዉ ; 3 = መወሰን አልችልም 2= አልስማማም; 1= በጣም አልስማማም

| ተ.ቁ | ተለዋዋጭ | **5** | **4** | **3** | **2** | **1** |
| --- | --- | --- | --- | --- | --- | --- |
| 1 | የመንግስትን ክልከላ ከቤት ባለመዉጣት ይተገበራል |  |  |  |  |  |
| 2 | ህዝብ በተሰበሰበበት መልኩ ሰርግ ይከናወናል |  |  |  |  |  |
| 3 | የቀብር ስነስርአት ህዝብ በተሰበሰበበት ሁኔታ ይከናወናል |  |  |  |  |  |
| 4 | የተለያዩ በአላት ማህበራዊ ፈቀቅታን ባልጠበቀ ሁኔታ ይከናወናል |  |  |  |  |  |
| 5 | በአምልኮት ቦታ ህዝብ ማህበራዊ ፈቀቅታን ባልጠበቀ ሁኔታ የአምልኮ ስርአት ይከናወናል |  |  |  |  |  |
| 6 | ህዝብ ትራስፖርት የተሳፋሪዎችን ቁጥር በግማሽ በመቀንሽ ያገለግላሉ |  |  |  |  |  |
| 7 | ህዝብ በሚሰበሰብበት ቦታ(ገበያ ፤መነሀሪያ) ሁልጊዜ የአፍነጫና አፍ መሸፈኛ እጠቀማለዉ |  |  |  |  |  |

**Questionnaire**

**WOLDIA UNIVERSITY**

**Faculty of Natural and Computational Science**

**Department of Biology**

**General Direction**

The purpose of this interview administered questionnaire is to gather information/data for the research purpose conducted under the title of **“Communities’ Knowledge, attitude and practice on SARS-CoV-2 Disease 2019 (COVID-19) in Woldia town, Northeast Ethiopia”**. This questionnaire consists of questions that focus on the personal profile of respondents, knowledge, attitude and practices of community on novel corona virus disease (covid-19) in Woldia town. The success of this study depends on the quality and trustworthiness of the responses given by the research participants (respondents). Hence, you are kindly requested to answer the questions carefully and come up with genuine information for the successful completion of the research. Finally, we would like to express our feelings of appreciation for your willingness to be part of this research work.

Thank you!

**Part one**: **Give answer for the following questions by using a tick mark “**✓**“in the box of your choice**

| **Demographic characteristics of the respondents** | | |
| --- | --- | --- |
| No | Variable |  |
| 1 | Age | ________ |
| 2 | Sex | M F |
| 3 | Marital status | Single |
|  |  | Married |
|  |  | Divorced |
|  |  | Widowed |
| 4 | Level of education | Illiterate |
|  |  | Ilitrate |
|  |  | Primary school complete |
|  |  | High school complete |
|  |  | Preparatory(TVET) |
|  |  | Diploma |
|  |  | Degree |
|  |  | Master |
| 5 | Occupation | Employed |
|  |  | Unemployed |
|  |  | Rerired |
|  |  | Business |

| **Knowledge, attitude and practice of the community about Covid-19** | | |
| --- | --- | --- |
| 6 | Source of knowledge about covid-19 | Mass media |
|  |  | Social media |
|  |  | Friends |
| 7 | Transmission mode | Through coughing and sneezing |
|  |  | Through direct contact with infected person |
|  |  | Through touching contaminated material |
| **8** | Clinical symptoms of COVID-19 | Fever |
|  |  | Dry cough |
|  |  | Sneezing |
|  |  | Difficulty to breathe |
| **9** | Pseudoscientific practices (using garlic, ginger, feto etc. as home remedies) are necessity for to confront **SARS-CoV-2** | Yes |
|  |  | No |
| **10** | Isolation and treatment of people who are infected with the COVID-19 are effective  ways to reduce the spread of the virus | Yes |
|  |  | No |
| **11** | COVID-19 will be successfully controlled | Yes |
|  |  | No |
| **12** | The government has been taken sufficient preventive measures to stop the spread of COVID-19 | Yes |
|  |  | No |
| **13** | Breast feeding can spread COVID-19 to new born babies | Yes |
|  |  | No |
| **14** | Children are not susceptible to COVID-19 | Yes |
|  |  | No |
| 15 | Frequent hand washing with soap | Yes |
|  |  | No |
| 16 | Frequent hand washing with sanitizer | Yes |
|  |  | No |
| 17 | Covering mouth and nose while coughing | Yes |
|  |  | No |
| 18 | Avoidance of touching eyes, nose and mouth | Yes |
|  |  | No |
| 19 | Practicing of social distancing | Yes |
|  |  | No |
| 20 | Wearing facemask in public or crowded places | Yes |
|  |  | No |
| 21 | Frequent cleaning of workplace surfaces | Yes |
|  |  | No |

**Part two: Government restriction strategy implementation by the community to prevent covid-19. Tick on the box by using tick mark “**✓**“in the box of your choice.** To what scale do you think the following variables help to combat Covid-19 pandemic? (Scale 1-5 where, 5= Strongly Agree (SA), 4 = Agree (A),3= Undecided (UD), 2 = Disagree (DA), and 1= Strongly Disagree (SD).

| No | **Variable** | **5** | **4** | **3** | **2** | **1** |
| --- | --- | --- | --- | --- | --- | --- |
| 1 | The communities implement the restriction by staying at home |  |  |  |  |  |
| 2 | Weeding ceremonies are implemented in the presence of overcrowded people |  |  |  |  |  |
| 3 | Funeral ceremonies are implemented in the presence of crowded people |  |  |  |  |  |
| 4 | Peoples celebrate holidays without implementing social distancing |  |  |  |  |  |
| 5 | Gathering of people in religious places (churches, mosques) is common |  |  |  |  |  |
| 6 | Reduction of the number of public transport passengers to 50% is well implemented |  |  |  |  |  |
| 7 | Regularly use face masks when you go to places with crowded people (e.g., market, bus station) |  |  |  |  |  |
|  | | | | | | |

Source: adapted from [9]
